# Diagnostic Value of Oblique Coronal and Oblique Sagittal Magnetic Resonance Imaging (MRI) in Diagnosis of Anterior Cruciate Ligament (ACL) Tears

**DOI:** 10.25122/jml-2018-0015

**Published:** 2018

**Authors:** Mohammad Ghasem Hanafi, Mohammad Momen Gharibvand, Razieh Jaffari Gharibvand, Hanon Sadoni

**Affiliations:** 1. Department of Radiology, Golestan Hospital, Ahvaz Jundishapur University of Medical Sciences, Ahvaz, Iran; 2. Department of Orthopedic Surgery, Ahvaz Jundishapur University of Medical Sciences (AJUMS), Ahvaz, IR Iran

**Keywords:** anterior cruciate ligament (ACL), orthogonal MRI, oblique-sagittal, oblique-coronal

## Abstract

**Introduction:** Tears of the anterior cruciate ligament (ACL) are common among young athletes and diagnosis may be difficult especially in the young population. Therefore, finding a new method to increase the correct diagnosis is necessary.

**Materials and Methods:** This double-blind prospective observational study was conducted on 51 patients with suspected ACL rupture. In this study, in addition to the standard protocols, the oblique-sagittal and oblique-coronal MRI were assessed and used in three different methods, including A method (orthogonal MRI protocol), B method (orthogonal MRI protocol and oblique-sagittal MRI), and C method (orthogonal MRI protocol and oblique-coronal MRI).

**Results:** In detecting both complete and partial rupture of ACL, B method had highest diagnostic accuracy (kappa = 0.338, P=0.001), and after that, C method had acceptable accuracy (kappa = 0.292, P=0.011). In addition, in detecting a partial rupture of ACL, B method (kappa = 0.5, P<0.001), and C method had acceptable accuracy (kappa = 0.361, P=0.006). Meanwhile, in detecting a complete rupture of ACL, B method had the highest diagnostic accuracy (kappa = 0.898, P<0.001), and subsequently A method had significant accuracy (kappa = 0.812, P<0.001).

**Conclusions:** Our results showed that the evaluation of ACL rupture by oblique-sagittal MRI in addition to orthogonal MRI protocol is accurate and with high sensitivity and specificity values. It allows to find abnormal images immediately with higher accuracy in the emergency department and more critically ill patients may benefit from the advantages of this imaging protocol.

## Introduction

Tears of the anterior cruciate ligament (ACL) are common among young athletes (approximately 200,000 cases per year in the United States [1]) and one of the most common orthopedic procedures [2], which can lead to chronic knee instability and joint function loss [3–5]. A recent meta-analysis shows that only 65% of patients with ACL injury return to their pre-injury level of a sport, which is due to the delay in starting appropriate treatment [6].

Magnetic resonance imaging (MRI) is a non-invasive imaging method with specific features for evaluating knee lesions such as good soft tissue contrast, multi-parameter and multi-range images and high spatial resolution [7]. Furthermore, MRI has high diagnostic value in evaluating the extent of the damage and damages to the related structures in knee lesions, but its diagnostic potential for tear or injury of ACL is limited and potentially fallible. [8] On the other hand, arthroscopy is another diagnostic method which allows direct visualization of all intra-articular structures. [9] But this method is relatively expensive and invasive [10], therefore, finding a new method or new procedure with high diagnostic value in evaluating ACL injury is needed. Tackling this issue, Kosaka et al. demonstrated that a new method was not needed, as MRI accuracy can be increased by using a different view of knee lesions such as ACL injury. They found that the additional use of oblique MRI improved the accuracy of diagnosis of ACL tear [11]. Moreover, Nenezic et al. showed that additional use of sagittal-oblique MRI has high accuracy for the evaluation of a complete rupture of the ACL. [12] To the best of our knowledge, there was an insufficient study regarding this issue in emergency patients suspected to exhibit ACL tears, so this study was designed to evaluate the diagnostic value of oblique coronal and oblique sagittal MRI in the diagnosis of ACL tears.

## Materials and Methods

This double-blind prospective observational study was conducted in the Radiology Department of Ahvaz Golestan Hospital, from January 2015 to March 2016. All MRI imaging was performed with the same devices. Inclusion criteria consisted of patients with knee trauma whose clinical examinations were likely to damage internal knee structures and were referred to the Radiology Department of Ahvaz Golestan Hospital as they required arthroscopy based on clinical conditions (decreased level of consciousness, hypoxemia, airway obstruction and manipulation of the airway and others). Exclusion criteria consists of patients with a history of arthroscopy, meniscectomy, previous restoration of ligaments, evidence of severe osteoarthritis (Grade 4) or fracture at the joint surface of the bones in knee X-ray, as well as those who had a contraindication of MRI (heart pacemaker, metallic foreign body such as metal sliver in the eye, aneurysm clip in brain, severe claustrophobia, and so forth) or a new trauma after MRI until arthroscopy. We also excluded the patients with uncompleted data.

## Participants

**Figure 1:**
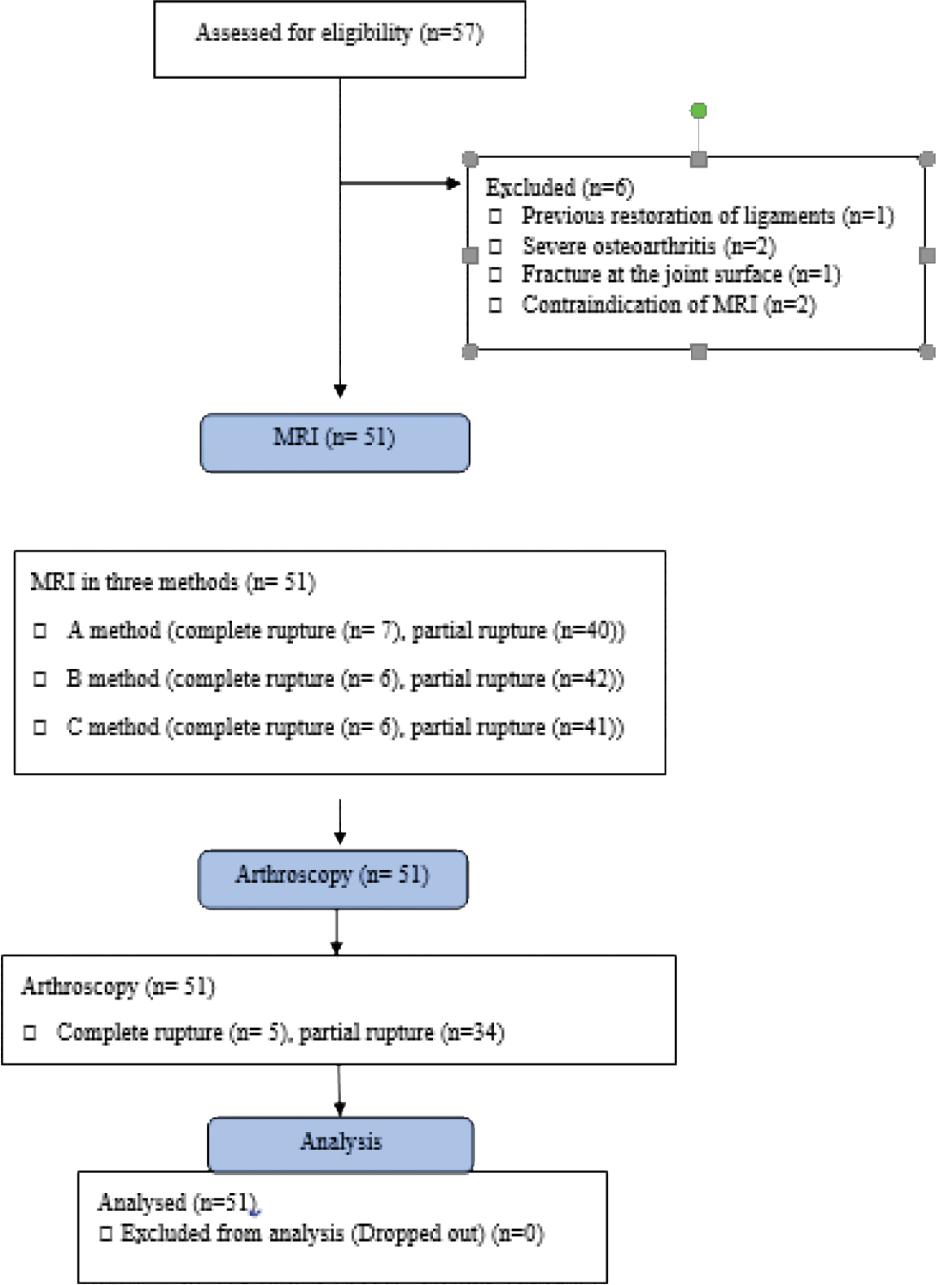
Study flowchart.

The study flowchart has been shown in figure 1. Fifty-one (out of fifty-seven) patients with an indication for arthroscopy, who had been diagnosed by an orthopedic surgeon and based on inclusion and exclusion criteria were included. The study received its ethics approval from the Ethics Committee of Ahvaz Jundishapur University of Medical Sciences.

Clinical examinations such as Lachman test, anterior and posterior drawer tests and pivot shifts tests were performed for all patients by an orthopedic specialist. In all patients, prior to arthroscopy, the MRI of the patients was reported by two radiologists and, if there was a difference in their reports, a third radiologist was asked.

Arthroscopy was performed in all patients with standard arthroscopic portals and arthroscopic findings were recorded in the operating theater

**Figure 2:**
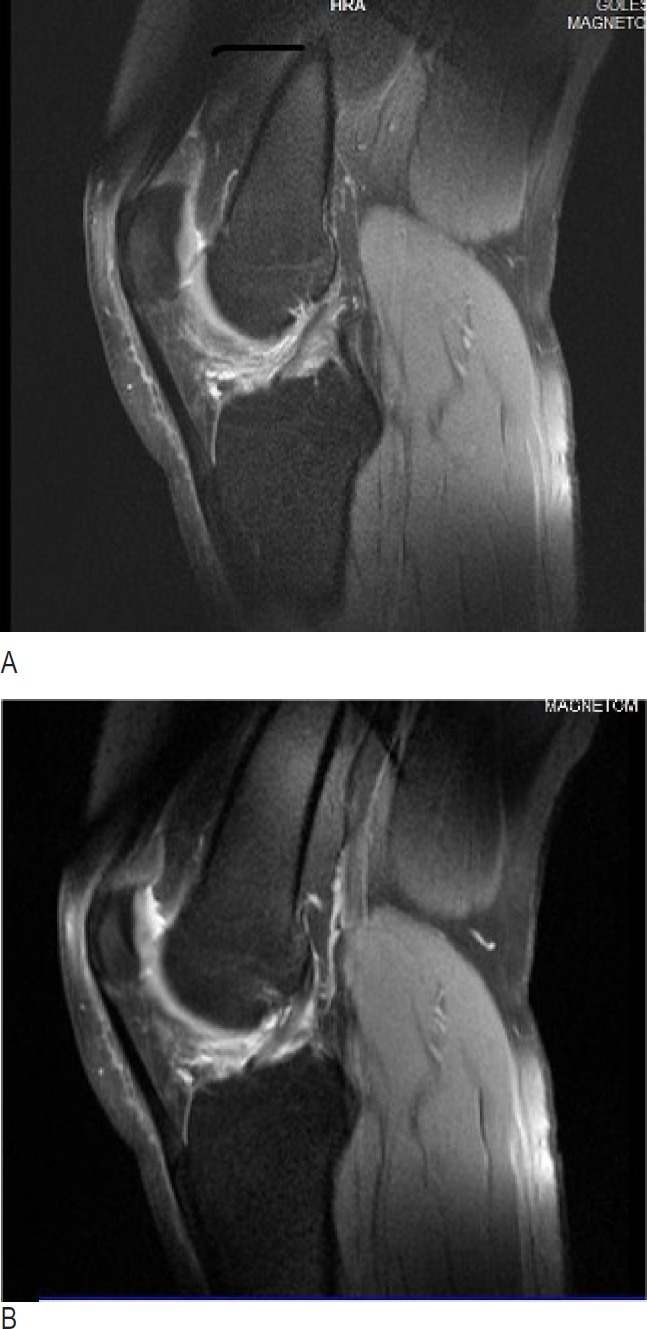
On standard orthogonal sagittal MRI (A) it is difficult to visualize the partial tear of ACL, while the partial tear on the oblique sagittal MRI (B) is obviously seen.

The MRI scanners used for all patients were equipped with a 1.5 Tesla (T) magnet. The orthogonal sagittal MRI performed by PD fat-saturated, and T2 in the sagittal, axial and coronal planes, as well as oblique-sagittal and oblique-coronal MRI, were also performed as PD fat-saturated (figure 2). The thickness of the slice was 3 mm and 1 mm. The oblique-coronal images were taken parallel to the intercondylar roof, and the oblique-sagittal images were taken from the outer border of the external femoral condyle in a standardized coronal image.

Demographic information and injury mechanism were also collected. In this study, in addition to the standard protocols, the oblique-sagittal and oblique-coronal MRI were assessed and used, and then MRI findings were reported separately using three methods (A-C) by two radiologists who were not aware of the patient’s clinical examinations and arthroscopic findings. The three methods include A method (orthogonal MRI protocol), B method (orthogonal MRI protocol and oblique-sagittal MRI), and C method (orthogonal MRI protocol and oblique-coronal MRI).

## Data Analysis

Data were analyzed and reported only for patients who completed the trial. Statistical analysis of data was performed using SPSS 22. Kappa coefficient was used in order to evaluate the level of agreement between different methods (A-C) and the gold standard (arthroscopy). The sensitivity, specificity, positive and negative predictive value and overall accuracy are necessary for all methods in evaluating complete rupture and partial rupture. The two-tailed p-value < 0.05 were considered significant.

## Results

The mean age of the studied patients was 37.01 ± 10.23 (19-58) year-old and 66.7 % of patients were male (34 cases). Six patients were dropped and finally, 51 patients completed the study.

Of 51 patients, 17.6% (9 cases) had fallen from a height, and 82.4 % (42 cases) had experienced direct trauma to the knee.

The findings of the two radiologists overlap as follows: A method 96.07% (kappa=0.898) (very good), B method 96.07% (kappa=0.882) (very good), and C method 100% (kappa=1) (excellent). As shown in Table 1, in detecting both complete and partial rupture of ACL, B method had the highest diagnostic accuracy (kappa=0.338, P=0.001), followed by C method with acceptable accuracy (kappa=0.292, P=0.011). However, both methods have low specificity, which was not good enough for conclusive results. Also, in detecting a partial rupture of ACL, B method had the highest diagnostic accuracy (kappa=0.5, P<0.001), followed by C method which had an acceptable accuracy (kappa=0.361, P=0.006). Moreover, in detecting a complete rupture of ACL, B method had the highest diagnostic accuracy (kappa=0.898, P<0.001), and A method had an acceptable accuracy (kappa=0.812, P<0.001).

**Table 1:** Sensitivity, specificity, PPV and NPV of AASI in detecting ACL rupture

Rupture	Method	TP	FP	TN	FN	SENS	SPE	PPV	NPV	LR+	LR-	Overall accuracy	Kappa	Sig.
Complete and partial	A	37	10	2	2	94.87%	16.66%	78.72%	50%	1.13	0.307	76.47%	0.15	0.194
	B	39	9	3	0	100%	25%	81.25%	100%	1.33	0	82.35%	0.338	0.001
	C	38	9	3	1	97.43%	25%	80.85%	75%	1.29	0.102	80.39%	0.292	0.011
Partial	A	30	10	7	4	88.23%	41.17%	75%	63.63%	1.49	0.285	72.54%	0.323	0.016
	B	33	9	8	1	97.05%	47.05%	78.57%	88.88%	1.83	0.062	80.39%	0.5	<0.001
	C	31	10	7	3	91.17%	41.17%	75.6%	70%	1.54	0.214	74.5%	0.361	0.006
Complete	A	5	2	44	0	100%	95.65%	71.42%	100%	22.98	0	96.07%	0.812	<0.001
	B	5	1	45	0	100%	97.82%	83.33%	100%	45.87	0	98.03%	0.898	<0.001
	C	4	2	44	1	80%	95.65%	66.66%	97.77%	18.39	0.209	94.11%	0.695	<0.001

A method: orthogonal MRI protocol, B method: orthogonal MRI protocol and oblique-sagittal MRI, C method: orthogonal MRI protocol and oblique-coronal MRI, TP: true positive, FP: false positive, TN: true negative, FN: false negative, SENS: Sensitivity, SPE: Specificity, PPV: Positive predictive value, NPV: Negative predictive value, LR+: Positive likelihood ratio, LR-: Negative likelihood ratio.

## Discussion

The results of our study showed that the B method (orthogonal MRI protocol and oblique-sagittal MRI) is the best method in evaluating complete and partial ACL rupture.

Kosaka et al. showed that the sensitivity, specificity, and accuracy of orthogonal sagittal and oblique coronal images for the detection of ACL remnant tissue were higher than orthogonal sagittal images (A method), orthogonal sagittal and additional oblique sagittal images (B method). Finally, they conclude that the use of oblique MRI improved the accuracy of diagnosis of ACL [11]. According to previous studies, orthogonal MRI protocol and oblique-sagittal MRI were better than other oblique images; however, in this study, we found that using orthogonal sagittal and oblique coronal images show much better results than oblique sagittal images. These differences may be due to different methods of study and the sample size. On the other hand, we found that oblique coronal images have high diagnostic accuracy after oblique-sagittal images. In another study performed by Kwon et al. it was demonstrated that the specificities and accuracies for additional oblique coronal images (method B), oblique sagittal images (method C), and oblique coronal and sagittal images (method D) were significantly higher than the specificities and accuracies for orthogonal images (method A). However, they found that there was no significant difference in the sensitivity, specificity, and accuracy for methods B, C, and D. [12].

Furthermore, Nenezic et al. showed that additional techniques (such as flexion and sagittal-oblique images) increase the diagnostic accuracy for the evaluation of a complete rupture of the ACL. Moreover, they found that sagittal-oblique images require a shorter scan time compared to the flexion technique due to a higher number of MRI slices that show the whole course of the ACL. [13]

Another study performed by Hong et al. demonstrated that the additional use of oblique coronal MRI images of the knee improves diagnostic accuracy in detecting ACL injury (kappa scores before and after use of oblique coronal images for first readings were 0.752 vs. 0.809 and for second readings were 0.784 vs. 0.843). [14] The results of this study were similar to ours; however, we found that the oblique-sagittal method has higher diagnostic value.

## Conclusions

Our results showed that the evaluation of ACL rupture by oblique-sagittal MRI in addition to orthogonal MRI protocol is accurate and with high sensitivity and specificity values. It allows doctors in emergency departments to find abnormal images immediately with higher accuracy and more critically ill patients may benefit from the advantages of this imaging protocol. Therefore, training and using this method for emergency physicians or radiologists should be considered in this and other countries. Further studies are required to confirm our findings.

## Conflict of Interest

The authors confirm that there are no conflicts of interest.

## Acknowledgments

This study was financially supported by Ahvaz Jundishapur Medical Sciences University, Ahvaz, southwest of Iran. We gratefully acknowledge the dedicated efforts of the investigators, the coordinators, the volunteer patients who participated in this study, and the Clinical Research Development Units (CRDU) of Golestan hospital.

## APPENDIX

### Statistical Terms and Definitions

• True positive: An ACL rupture that had been diagnosed correctly (A)
• False positive: Another diagnosis except for ACL rupture that had been diagnosed as ACL rupture (B)
• True negative: Another diagnosis except for ACL rupture that had been diagnosed correctly (D)
• False negative: ACL rupture that had been misdiagnosed (C)
• Sensitivity: The percentage of correctly diagnosed ACL ruptures as a proportion of all ACL ruptures that were diagnosed (A/A+C))
• Specificity: Probability of a negative test result in an individual without the condition D/(B + D)
• Positive predictive value: The probability of having the condition when the test result is positive A/(A + B)
• Negative predictive value: The probability of not having the condition when the test result is negative(D/(C+D))
• Accuracy: The percentage of correctly diagnosed ACL ruptures or another diagnosis as a proportion of all diagnosis (A+D)/ (A+B+C+D)
• The likelihood ratio of a positive test result (LR+): The number of times that a patient with positive test results will have ACL rupture; it is calculated by dividing sensitivity by 1-specificity.
